# Epidemic model for information diffusion in web forums: experiments in marketing exchange and political dialog

**DOI:** 10.1186/s40064-016-1675-x

**Published:** 2016-01-22

**Authors:** Jiyoung Woo, Hsinchun Chen

**Affiliations:** Graduate School of Information Security, Korea University, Anam-ro, Seoul, Korea; Eller College of Management, University of Arizona, 1130 E. Helen St, Tucson, AZ USA

**Keywords:** Information diffusion, Epidemic model, Contagion, Web forum, Social media

## Abstract

As social media has become more prevalent, its influence on business, politics, and society has become significant. Due to easy access and interaction between large numbers of users, information diffuses in an epidemic style on the web. Understanding the mechanisms of information diffusion through these new publication methods is important for political and marketing purposes. Among social media, web forums, where people in online communities disseminate and receive information, provide a good environment for examining information diffusion. In this paper, we model topic diffusion in web forums using the epidemiology model, the susceptible-infected-recovered (SIR) model, frequently used in previous research to analyze both disease outbreaks and knowledge diffusion. The model was evaluated on a large longitudinal dataset from the web forum of a major retail company and from a general political discussion forum. The fitting results showed that the SIR model is a plausible model to describe the diffusion process of a topic. This research shows that epidemic models can expand their application areas to topic discussion on the web, particularly social media such as web forums.

## Background

Social media such as blogs, discussion forums, and social networking sites provide new channels for individuals to share information and express their opinions. The characteristics of social media, such as rich representation, low cost, easy accessibility, and rich user interaction, have created new opportunities for marketers and politicians to leverage social media for their businesses. The prevalence of social media enriches information that people share and accelerates its diffusion between them. The information diffusion process is a successive result by which people influence one another over a time period (Kleinberg 2008). The social interaction on the web has become a new source of information diffusion, which was only available to traditional mass media in the past.

As the influence of social media becomes more evident, understanding the mechanisms and properties of information diffusion through these new publication methods is important for political and marketing purposes. The word-of-mouth propagation through blogs, email, and product review forums has been studied for marketing purposes. Researchers have also studied how political messages diffuse on the web through personal blogs or information-sharing websites. However, few studies have focused on the more restructured and spikey interactions expressed in public web forums. Web forums are important and popular for marketing exchange and political dialog. Unlike blog or email networks that are dominated by a few bloggers or known acquaintances, web forums allow opinions to be freely formed and spread in society. Anyone can begin a new thread of discussion and anyone can participate freely and equally. People who have common interests express and discuss their opinions and affect each other. Among all social media, web forums are promising for modeling information diffusion. In this article, we propose a new extension of the SIR model for information diffusion on web forums. Our design expands significantly from the baseline SIR epidemic model for information diffusion. This paper is organized as follows. “Related work” section summarizes previous research on diffusion modeling, focusing on information diffusion, and presents previous studies that support opinion contagiousness. In “Information diffusion model in web forums” section, we present the SIR model, develop the analogy between the epidemics and topic diffusion in the web forum and propose a new extension of the SIR model in the web forum. “System design: SIR for web forums (the SIRW system)” section presents the system design of diffusion modeling and elaborate the system components. Experiment results are reported in “Experiment result” section. Discussion including pros and cons of this research and conclusions and future directions are presented in “Discussions” and “Conclusions” sections.

## Related work

Diffusion refers the process whereby a phenomenon of interest (e.g., information, innovation, or disease) spreads from one to another (Cliff and Haggett 2005). Related to human, diffusion is a long history of the research topic in epidemiology and sociology domains. Rich literature deals with information diffusion between people. As the social media became an everyday communication channel between people, various online social networks are formed, and they reflect the real-world social network. As the social network draws much attention from researchers, the diffusion process on the online social network became an ongoing research topic. Due to similar patterns in the spread of epidemics and social contagion processes, most research adopts the same theoretical principles for epidemics in describing the information diffusion. The mainstream theory that explains the epidemic defines the disease diffusion as the spread of memes of infection (Blackmore 2000). Thus, most epidemic models are mainly based on the contagion through the interaction between people. Micro-level epidemic diffusion models firstly set the population structure and build non-linear differential equations that describe the status of change in the population class. These micro-level models are named as equation-based models. Equation-based models (EBMs) operate on global laws defined by the equations and applied to all members of the compartment. The underlying assumption of EBMs is that the population is homogeneous and is governed by holistic rules. They assume that people have a constant contact rate and are infected by a disease that has a unique transmission rate, and recover at a certain rate. The stochastic model uses the concept of independent and identically distributed objects, but it still describes the diffusion process with holistic rules (Bobashev et al. 2007). Using diffusion models, we can understand how new diseases, information, or products spreads, to predict their success or failure in the early stages, and to increase or reduce the chances of diffusion. Early stage models do not reflect the social network underlying in the population. In some disease, this assumption fits well since the disease can diffuse through indirect interaction as well as direct contact. The model that assumes the homogeneous mixing between individuals, in other words, random contact is named the population model. The population model divides a population into classes that reflect the status of individuals in the population. The network-based model considers the network in which diffusion happens and focuses on the effects of network properties in the diffusion process. Diffusion studies have been supported by recent findings from real-world networks, including social networks and their topological features (Barabási and Albert 1999). The SIR model (Kermack and McKendrick 1927), a representative epidemic model, has three compartments of susceptible, infective, and recovered. The model expresses the status change of three compartments using differential equations. The independent cascade model (ICM) (Goldenberg et al. 2001), which is widely adopted in describing the information diffusion on online social networks, is a special case of the SIR model reflecting the network structure of the population. The first study on information diffusion modeling using epidemic models has been made using the study on the spread of scientific ideas. Goffman and Newill (1964) developed the analogy between the adoption of scientific information and the spread of infectious disease. Bettencourt et al. (2006, 2008) developed Goffman and Newill’s (1964) work by proposing the competency model that describes the diffusion process of two competing theories. Epidemic models were also tested to rumor propagation (Kawachi 2008). Epidemic models have also been used for modeling financial information diffusion. Fan (1985) proposed ideodynamics model that embeds people’s contact and content characteristics. Fan and Cook (2003) added the sentiment of mass media content to model consumer sentiment about the economy. Shive (2010) modified the SIR model to predict the buying and selling of a stock by adding situational determinants such as total trade amount, return on investment, and income level to the social interaction. Shtatland and Shtatland (2008) approximated the SIR model into a first-order autoregressive model for the financial outbreak. From a marketing perspective, online word-of-mouth has become a new topic of diffusion modeling. Goldenberg et al. (2001) studied the underlying process of word-of-mouth on the network. Song et al. (2007) proposed the rate-based information flow model using the Markov chain and applied it to recommendation systems. Bampo et al. (2008) applied the SIR model to various ideal networks to measure the efficiency of email marketing campaigns. As online information diffusion has become a major topic for diffusion studies due to the growth of email, the web, and social media, epidemic models have been applied to modeling of information diffusion on the web. New attempts to apply diffusion models to email, blogs, and forums have emerged. Many studies examine information diffusion in the blogsphere. Gruhl et al. (2004) defined the characteristics of diffusing topics in the blog and proposed a method for estimating the transmission probability for ICMs. Saito et al. (2008) used expectation maximization to estimate the transmission probability for ICMs. Leskovec et al. (2007) suggested the cascade generation model under the SIS framework with fixed transmission probability.

The research on web forums differs from that on blogs in that it focuses on the diffusion vector such as topics, news, and documents. Kubo et al. (2007) showed the analogy between the disease propagation model, the SIR model, and posting data in web forums. Woo et al. (2011) adopted the SIR model to model violent topic diffusion in the Jihadi forum. Woo and Chen (2012) extended Kubo’s work incorporating the new media effects. They focused on how new media effects can be reflected in the SIR model. Kubo et al. (2007), Woo et al. (2011) and Woo and Chen (2012) performed modeling the post dynamics with the SIR model and without the sound analogy between information diffusion and epidemic, logical arguments and interpretation of the proposed model. These works viewed the information diffusion in post-level not author-level that is more appropriate for modeling the authors interaction and thereby contagion of a topic. The post-level works consider the post as the carrier and itself as the result of diffusion, this results that susceptible class does not explain clearly. To overcome these shortcomings, we develop the sound analogy between information diffusion in the web forum and epidemic model, and propose the system design to examine the information diffusion in the web forum.

In recent, online social networking sites like MySpace, Facebook, Flickr, and Twitter, have become popular ways to share and receive information. A complete digital record provides an opportunity to observe the information diffusion in online social media. Especially Twitter, which is a micro-blogging system, become popular and has received much attention from industry and academic fields. Romero et al. (2011) identified the differences in the mechanics of information diffusion across topics: idioms, political hashtags, and complex contagion on Twitter. Sakaki et al. (2010) proposed an algorithm to monitor tweets and to detect a target event. Their model is based on a spatial and temporal model, and it tracks and models the information diffusion related to an event. Lerman and Ghosh (2010) performed the measurement study on dynamics and distribution of fan votes in Twitter. Toole et al. (2012) adopted the susceptible-infected-susceptible (SIS) model to model the adoption of Twitter and proposed the modified model to reflect the presence of geographic and media influences. Myers et al. (2012) presented a model in which information can reach a node via the links of the social network or through the influence of external sources. They then applied the model to the emergence of URL mentions in the Twitter network. Especially, they pointed that information diffusion does not happen merely by the contagion and argued that diffusion model should reflect the mass media effect. Sun et al. (2009) performed the empirical investigation of information diffusion through a large social network through Facebook. They used the regression model to identify affecting factors on large chain diffusion. Cha et al. (2009) analyzed large-scale traces of information dissemination in the Flickr social network and found that even popular photos spread slowly through the network. Tang et al. (2014) models information diffusion through hashtag in Sina Weibo, micro-blogging such as Twitter using the susceptible-infected (SI) model. Wang et al. (2015) proposed the emotion-based SIS model for and showed that it outperforms SIS model in describing information diffusion with Twitter data. Liu and Zhang (2014) proposed a dynamic susceptible-infected-recovered (SIR) model considering dynamic rewiring network in which people can break links and reconnect to their second-order friends. Jalali et al. (2016) presented a dynamic model to quantify the core mechanisms of petition diffusion including invitation, which is the contagion factor, interest, awareness, forgetting, sharing and reminding. Above mentioned research on information diffusion through social media has mainly focused on blogs email, Twitter and how the network structure affects information flow. However, blog connections are heavily dominated by a few bloggers, email limits itself to known acquaintances and Twitter allows information diffusion through the follower network. Only web forums provide a truly open and freely accessible platform for information diffusion. Research on information diffusion in web forums can analyze the rich data in the forums topics and messages, rather than just the network structure. We highlighted key papers on information diffusion in terms of the model species, applications, and contributions in Table 1.

**Table 1:** Previous research on information diffusion

Key papers	Model specification	Applications	Contributions
Goffman and Newill (1964)	SIR, SIS	Scientific theory	The first analogy development between information and disease diffusion
Kawachi (2008)	SIR-variants	Rumor	The novel model with offsetting effect
Fan (1985)	SIR	Financial information	The novel model with content characteristics ideodynamics model
Shive (2010)	SIR	WOM of stock	Novel model with corporate financial information
Shtatland and Shtatland (2008)	SIR	Financial information	Outbreak detection using the diffusion model
Goldenberg et al. (2001)	SIR	Word of mouth (WOM)	The network effects on WOM
Bampo et al. (2008)	ICM	WOM	The network effects on WOM
Gruhl et al. (2004)	ICM	Blog	The empirical test
Saito et al. (2008)	ICM		The method to estimate infection rate
Leskovec et al. (2007)	Network SIS	Blog	The empirical test
Kubo et al. (2007)	SIR	Web forum	The analogy development between topic diffusion in the web forum and disease spread
Toole et al. (2012)	Network SIS	Twitter	The novel model with geolocation information, the empirical test
Myers et al. (2012)	ICM	Twitter	The novel model with external effect
Tang et al. (2014)	Network SI	Chinese Twitter	The empirical test
Liu and Zhang (2014)	ICM	Syntactic data	The novel model with rewiring friendship
Wang et al. (2015)	Network SI		The novel model, emotion-based spreaderignorantstifler (ESIS) model

## Information diffusion model in web forums

### SIR model in web forums

**Fig. 1:**
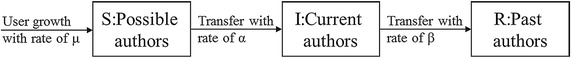
Transition diagram of the SIR model in web forums

The dynamics underlying the diffusion of ideas and opinions have many similarities to disease infections (Bettencourt et al. 2006). Disease infection spreads through contact; it starts when a few individuals are infected. Each infected person then has contact with others. Individuals who have had contact with infected people become infected themselves at a certain rate. Infected individuals come into contact with additional individuals. This infection process continues until no more susceptibles or infectives exist. The SIR-based epidemic models represent the cycle of disease in three phases, Susceptible, Infective, and Recovered, which are connected to each other with a certain infection rate ($\alpha$) and recovery rate ($\beta$) (Kermack and McKendrick 1927). When the population varies over time, we need to consider the population growth or loss with a certain rate (*µ*).The SIR model builds up system equations in forms of differential equations. In a web forum, the posting action causes instant contagion and reaction to/from other users. The epidemic model is analogous to the diffusion process of topical discussions on the web. An initiating author begins a discussion on a topic by posting a thread; the author becomes an infective. The users who have a certain level of interest in a topic (susceptibles) will read and post comments on the thread. Some commenters and readers will post other threads, thereby infecting others with information in their posts. After a certain period, some authors may lose interest and stop participating in discussions, thus losing the power to influence others. This interactive process leads to the flow of discussion on a topic from one author to another. When this influence power is significantly high, the diffusion curve embeds an exponential rise, turnover, and decay. Otherwise, the diffusion curve declines without a significant rise (Allen et al. 2008). The epidemic model of information diffusion in the web forum is depicted in Fig. 1.

Unlike the email and blog that provide a major channel of information diffusion, which is the user’s social network, the web forum where anyone in the community participates in the discussion does not provide a major channel of information diffusion. Since posts are exposed to any users in the web forum, the contact can occur between anyones. Thus, the population-based epidemic model is not an appropriate for the web forum rather than the network-based model extensively explored in the email and blog. Goffman and Newill (1964) firstly proposed to the analogy between information diffusion and epidemic model. They defined the intellectual epidemic as that people are susceptible to certain ideas and resistant to others, once an individual is infected with an idea and transmit it to others. They adopted a population-based model for an intellectual epidemic where anyone can be infected with an idea. Their model was applied to the physical theory adoption. Based on Goffman and Newill’s (1964) analogy, we developed the analogy between the compartment SIR model and the topic diffusion in the web forum. The elements of the SIR model, defined in the context of epidemics and topic diffusion in web forums, are described in Table 2.

**Table 2:** The analogy between epidemics and topic diffusion in the web forum

Elements of SIR model	Epidemics	Topic diffusion in web forums
What flows	Disease	Idea/topic (keywords)
Susceptible: S(t)	People who can have contact with an infective and possibly will become infected	Possible authors (including commenters) who might read posts on a topic
Infective: I(t)	People who have a disease and possibly will infect others	Current authors who write posts on a topic
Recovered: R(t)	People who recover from a disease and lose the power to infect others	Past authors whose posts lose influence toward others
Infection rate: *α*	The probability of transmission in a contact between an infective and a susceptible	The probability of writing a comment or thread after reading posts on the topic
Recovery rate: *β*	The probability that the infective becomes recovered	The probability that posts lose infectivity
Recruitment rate: *μ*	The proportional increase rate of the population	The proportional increase rate of author pools
Carrying capacity: K	The maximum population that the environment can support	The highest value of the total authors that a topic can recruit

### Mathematical formulation

In the web forum context, S(t) is the number of possible authors who might have an interest in a topic at time t. I(t) is the number of authors who write posts on the topic during the same period. R(t) is the number of authors whose posts lose infectivity to others on a topic. $\alpha ,$ infection rate, indicates how many possible authors will be infected per contact between an infective and a susceptible. $\beta ,$ recovery rate, indicates how many infective authors per infective recover during a unit time. Since infection occurs through a contact between susceptibles and infectives, the increase of infective authors is determined by the effective contact between possible authors and current authors. The number of authors who become infected from susceptibles is described as the product of $\alpha$, S(t), and I(t). We express the compartments and parameters of the model in Eq. (1) (Table 2).

1 $$\begin{aligned}&s(t)={{\delta S}\over {\delta t}}\;\text { at time t}\nonumber \\&i(t)={{\delta I}\over {\delta t}}\;\text { at time t}\nonumber \\&r(t)={{\delta R}\over {\delta t}}\;\text { at time t} \end{aligned}$$

*S*(*t*) the number of future authors at time t, *I*(*t*) the number of current authors at time t, *R*(*t*) the number of past authors at time t, $N(t)=S(t)+I(t)+R(t)$ the total population size, $\mu$ the population growth rate, $\alpha$ the infection rate, $\beta$ the recovery rate.

The decrease of infective authors is determined by the recovery rate and the number of infective authors. The total change of the number of infectives is the sum of the product of $\alpha$, S(t), and I(t) and the product of $\beta$ and I(t). This results in Eq. (2).

2 $$i(t)=\alpha S(t)I(t)-\beta I(t)$$

The decrease of infectives outflows to the recovery class, so the derivative of the recovery class is expressed as shown in Eq. (3).

3 $$R(t)=\beta I(t)$$

The increase of infectives comes from the susceptible class. The plausible model that reflects the growth of forum users and the growth of users’ interests must allow the total population to vary in time. The logistic growth is a common model of population growth where the rate of reproduction is proportional to both the existing population and the number of available resources. In the logistic growth, population grows with population size and there is an upper limit, called the carrying capacity, to the population size. Because incoming users are initially susceptible, we add the logistic growth term in the differential equation of susceptible class. From the carrying capacity, K, we can estimate the maximum number of authors in a topic. The logistic growth of susceptible authors is represented as the rate of $\mu \left( {K-{N(t)}\over {K}}\right) N(t)$. Mathematical equations are formulated as follows:

4 $$s(t)=\mu \left( {K-{N(t)}\over {K}}\right) N(t)-\alpha S(t)I(t)$$

We evaluated the proposed model by data fitting. The evidence of the model’s success in fitting historical sales data lends credibility to the model’s basic structural soundness and its utility as a forecasting device (Wan and Xiao 2010). The Non-linear Least Squares method is considered suitable for solving differential equations in many diffusion studies. It refines the model parameter by successive iterations that aim to minimize the residuals.

5 $$\underset{\theta \in F}{{\text {argmax}}}\; J(\theta )=\sum _{i=1}^{n}\left( I_{i}-\hat{I}\left( t_i,\theta \right) \right) ^2$$

$I_{i}$ observed value at point i (from real data), $\hat{I}_{i}$ expected value at point i (from the model output), *F* feasible set, $\theta$ parameter set to be estimated.

Equation (5) is the objective function for the iterative parameter estimation. I(t) is an observation variable, in our case, it is the number of authors who participated in the discussion on a topic. The parameter set is composed of $\alpha$, $\beta$, and $\mu$, and they are optimized to minimize the above objective function. The parameters should be non-negative and be <1 except $\mu$. The initial condition of S(0), the author pool, K, are estimated. S(t), I(t), and R(t) are iteratively updated following Eqs. (2)–(4).

## System design: SIR for web forums (the SIRW system)

### Data collection

**Fig. 2:**
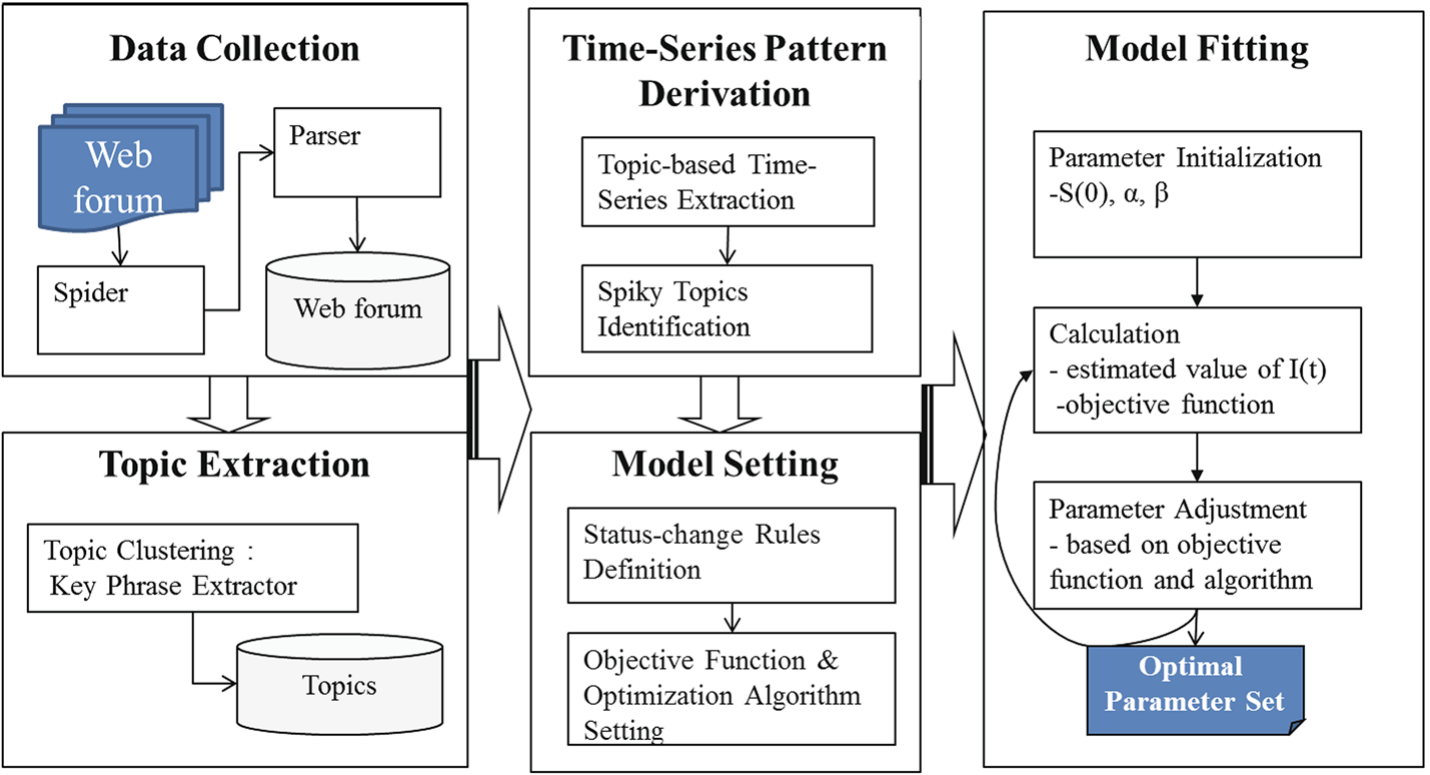
SIRW system design

We developed an integrated and novel system design as shown in Fig. 2. To collect data from the web forum, we developed a forum spider and parser. The spider crawls each HTML page in the web forum. Because a web forum consists of web pages linked to each other by hyperlinks, the spider can traverse the web to collect the page data. The crawler is composed of the page crawler and the content crawler. The page crawler retrieves the URLs of the pages that contains the contents in the web forum. Current page links next pages, so the crawler follows the links embedded in the current page, retrieves the link pointing the next page and moves the cursor to the next page. The crawler repeats this process until it reaches to the end page. The content crawler the content threads and replies. Each front page shows the thread list and each thread links the web that contain the contents of the thread and the replies. The parser uses the regular expression that finds specific patterns in a text. HTML files use a unique tag to express certain information. We developed the parser to extract key data fields such as thread identity, user identity, and messages within the HTML files.

### Topic extraction

To derive keywords, we performed topic clustering using a probabilistic topic model, especially latent Dirichlet allocation (LDA) (Blei et al. 2003). A topic modeling technique is designed to automatically uncover thematic structure in a large collection of unstructured text (McCallum 2002). According to topic modeling, a document is defined as a mixture of various topics. A topic is defined as a set of words that frequently occurred together. The probabilistic model assumes that documents are mixtures of topics, where a topic is a probability distribution over words. The probabilistic topic model works to find out the best set of words and to explain the shown-up words in documents. The probabilistic topic model specifies a simple probabilistic procedure by which documents can be generated. To make a new document, one chooses a distribution over topics. Then, for each word in that document, one chooses a topic at random according to this distribution and draws a word from that topic. This process is inverted using statistical methods inferring the set of topics that were responsible for generating a collection of documents (Blei et al. 2003). The adopted probabilistic model clusters messages according to probable keywords. In LDA, the topic distribution is assumed to have a Dirichlet prior that is often used as prior distributions in Bayesian statistics. Dirichlet priors make the statistical inference simple and output more reasonable mixtures of topics in a document (Steyvers and Griffiths 2007).

The algorithm works in an iterative way as follows. First, we set the number of topics. Then algorithm assigns the every word to a temporary topic according to a probability distribution. Since each word is assigned in a somewhat random manner, a word shown up more than once in different documents can be assigned to different topics. Then the algorithm takes an iterative topic assignment as follows. A word is assigned to the topic where this word is the most prevalent and a document is assigned to a topic where the words in the document are mostly assigned. After the iteration converges, topic modeling is done. For topic clustering, we used Mallet (McCallum 2002) that implements LDA for large-scaled unstructured data. In Mallet, we need to address the semantic meaning of topics by considering the words in the topic. When the number of topics is set to too low, words in a topic are general and a topic includes the words that are not close semantically. When the number of topics is set to too high, words in a topic become too specific, and words distribute to various topics. This causes semantic overlapping of topics. Thus, we varied the number of topics and examined whether topic modeling generates proper semantic clusters. We incrementally set the number of topics by ten and observed the semantic generation.

We defined key topic as the topic cluster that has a significant volume of posts and authors, is discussed actively at current and is composed of meaningful keywords. We cluster messages in a large number of classes and select meaningful topic clusters with keywords that reflect user needs. We selected bigrams from extracted keywords to derive topics that are meaningful and have significant volume.

### Time-series patterns

In the time-series pattern derivation module, the numbers of distinct authors are derived by aggregating postings that include a topic in a time frame. According to Gruhl et al. (2004), topics are classified into chatter topics and spikey topics. The chatter topic is defined the topic of ongoing discussions whose subtopic flow is largely determined by decisions of the authors. The spikey topics are high-intensity discussions of real-world events that are relevant to the topic (Gruhl et al. 2004). The spikey topic induces sharp rises in postings. They looked at topic occurrences and defined a spike as an area where the posts on a given day exceeded $\mu + 2\sigma$ (Gruhl et al. 2004). We followed the same manner. We analyzed the time-series patterns of key topics to exclude chatter topics. Chatter topics with ongoing patterns were also excluded from the analysis because topics without epidemic patterns were not considered to be contagious and cause contagion between users. Spiky topics, which are topics that draw interest, i.e., are infectious, are then selected. In the next step, interaction rules, observation variable, estimation variable, objective function, and parameters to be estimated are defined as mentioned in “Information diffusion model in web forums” section.

### Model fitting

In the model fitting step, data are tested for the model using a user-defined optimization algorithm. The Genetic Algorithm (GA) is employed as the optimization algorithm for parameter estimation. The wide range of methods to implement GA has been developed. We outlined the major procedures and selected algorithm in each procedure. First, a fitness function that indicates how well the current population fits the objective function is determined using the linear-ranking algorithm of Baker (1987). The fitness function affects the population selection. To reproduce the population in each generation, the selection method that extracts chromosomes from population should be fixed. In this work, we used roulette wheel selection (Golberg 1989). The crossover routines recombine pairs of individuals with given probability to produce offspring. Single-point (Booker 1987) is used. For mutation operation, real-value mutation (Mühlenbein and Schlierkamp-Voosen 1993) is adopted.

We evaluate the SIR model regarding the goodness of fit including: mean squared error (MSE) and R-square, defined in Eq. (6).

6 $$\begin{aligned}&MSE={1\over n}\sum _{i=1}^{n}\left( I_{i}-\hat{I}( t_i,\hat{\theta }) \right) ^2 \nonumber \\&R{\text {-}}square=1-{{\sum \nolimits _{i=1}^{n}\left( I_{i}-\hat{I}( t_i,\hat{\theta }) \right) ^2}\over \sum \nolimits _{i=1}^{n}\left( I_{i}-\bar{I}\right) ^2} \end{aligned}$$

$I_{i}$ the number of infectives at time i, $\bar{i}$ the average of $I_{i}$, *n* the number of samples, *i* time point, $\theta$ the estimated parameter set.

## Experiment results

We examined the proposed model in a marketing exchange and political dialog. Two web forums that have numerous active participants were studied. For the marketing exchange, we chose the “Yahoo! Finance—Walmart message board”, which provides a longitudinal dataset covering 10 years where various stakeholders actively express their opinions on various topics relevant to Walmart. The political forum, “US Politics Online—Breaking News in Politics” was chosen because it has many active authors and it covers many general political topics.

- *Yahoo! Finance* Walmart message board (January 1999–June 2008; 139,062 threads; 441,954 messages; 25,500 authors).
- *US Politics Online* Breaking News in Politics (May 2005–March 2010; 2192 threads; 130,850 messages; 1124 authors).

### Marketing exchange: Walmart forum

**Fig. 3:**
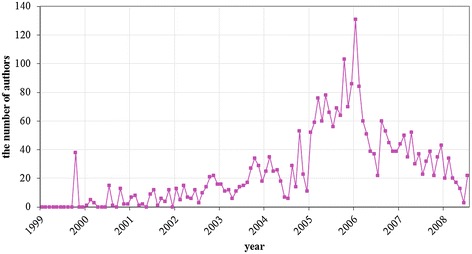
Trend of author participation

The number of author participation is depicted in Fig. 3. In the early stage of forum creation, there is a peak of author participations. Also, there is a major peak in 2005. We examined major topics in two peaks to analyze which topics lead author participations.

**Fig. 4:**
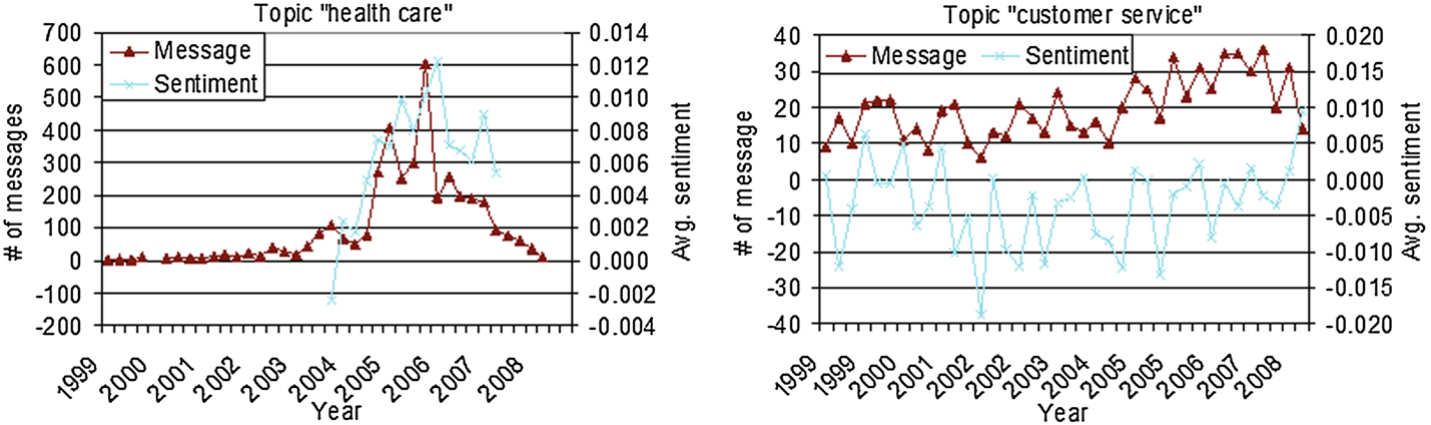
Spikey topic vs. chatter topic

According to Gruhl et al.’s (2004) research, the collection of posting about the topic has different patterns and can be categorized into chatters and spikes. Chatter topic is the ongoing discussion and spikey topic is the high-intensity discussion. In the web forum, we could also observe two types of topics. In Fig. 4, the left panel shows a spikey topic and the right panel shows a chatter topic.

To derive topics, we performed topic clustering using Mallet. The number of clusters is set to minimize the overlap of keywords between clusters. For the Walmart forum, we grouped threads into 50 clusters. From 50 clusters, chatter topics that do not cause a peak were excluded. Major spiky topics embed significant volumes the SIR analysis were included. Then, we selected topics that cause two major peaks in the author participation pattern. The major topics in the Walmart dataset encompass customer-related, investor-related, and employee-related topics as shown in Table 3. The keywords in the table are representative words for each topic cluster.

**Table 3:** The major topics and keywords in the Walmart forum

Topic group	Topic	Keywords
Investor	Stock price	Growth, share, earnings, price, stock, market
	Sales	Sales, percent, quarter, increase, fiscal, earnings, expected, results
Customer	Low price	Prices, low, economy, consumer, cost, market
	Shopping convenience	Shopping, items, manager, shoppers, service, line, door, experience
Employee	Healthcare	Healthcare, employees, insurance, medical, plan
	Labor law	Labor, illegal, federal, laws, violations, rights
	Wage	Pay, wages, benefits, employees, hour, working paid average hours, minimum, poverty, paying

**Fig. 5:**
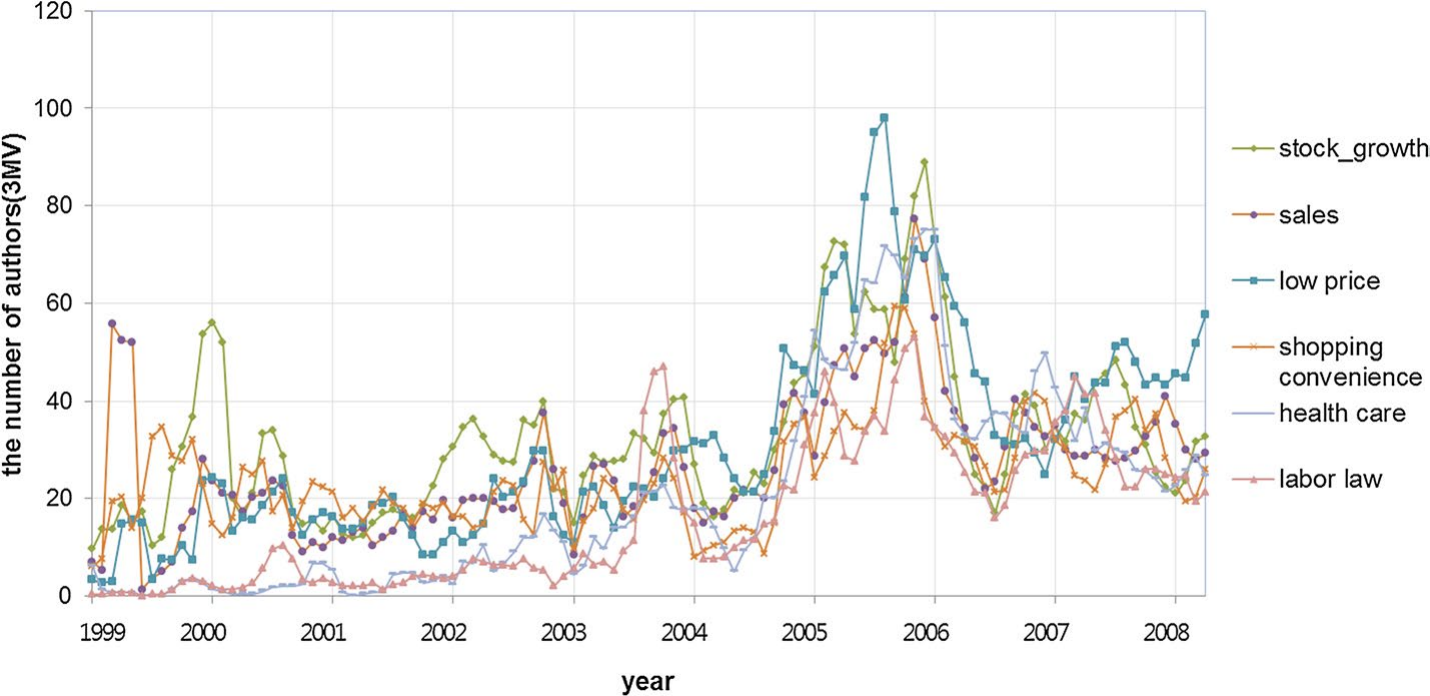
The time-series patterns of selected Walmart topics

The number of distinct authors, including commenters, whose posts belong to the threads where users still post in was counted monthly for the topics over the major outbreak periods. The time-series of selected topics are displayed in Fig. 5. We used the aggregated 3-month moving average value to reduce the effect of time-series fluctuation.

Table 4 summarizes the results of parameter estimation of the SIR model in the Walmart forum. The estimation samples consisted of 30 monthly observations for the second peak of author participation. The sample set includes the data from April 2004 to September 2006 when the selected topics have major outbreaks. The SIR model performed well in modeling major topics with R-square ranging from 0.52 to 0.75.

**Table 4:** Parameter estimation results on the Walmart forum

Topic	MSE	$R^{2}$	S(0)	$\alpha$	$\beta$	$\mu$	K
Stock price	5.28E+03	0.6198	163	0.0045	0.6798	0.1226	1384
Sales	2.72E+03	0.6320	100	0.0081	0.7270	0.1388	997
Low price	3.64E+03	0.7262	122	0.0059	0.7506	0.1419	1401
Shopping convenience	1.98E+03	0.6433	116	0.0078	0.7914	0.1230	1000
Healthcare	3.83E+03	0.7190	116	0.0065	0.7677	0.1361	1200
Labor law	1.16E+03	0.7510	89	0.0088	0.7433	0.1324	800
Wage	6.55E+03	0.5209	100	0.0053	0.6000	0.1524	950

**Fig. 6:**
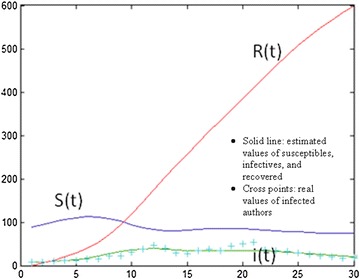
The estimated and real values of the SIR model on the topic of labor law

For the topic of stock price, for example, the optimal values of the parameters were estimated at 162.5 for S(0), 0.0045 for $\alpha$, and 0.68 for $\beta$. This implies that the number of forum users who might have an interest in the topic of stock price and possibly become authors would be approximately 163 out of about 312 total forum authors at April 2004; the number of infected authors for 10,000 susceptibles would be 45 per month; and the number of infected authors who would recover per 100 infectives during a month would be 68. The topic of sales has a lower number of initial possible authors, higher infection rate, and lower recovery rate than the topic of stock price; which implies that stock price is a less spiky topic than sales. The low price topic in customer-related topics and the wage and healthcare topics in employee-related topics are less spiky than others. The numbers of susceptibles, infectives, and recovered at successive times are derived by solving differential equations with the optimal parameter set. Figure 6 displays real values of infected authors and estimated values of susceptibles, infectives, and recovered on the labor law topic.

### Political dialog: political web forum

**Fig. 7:**
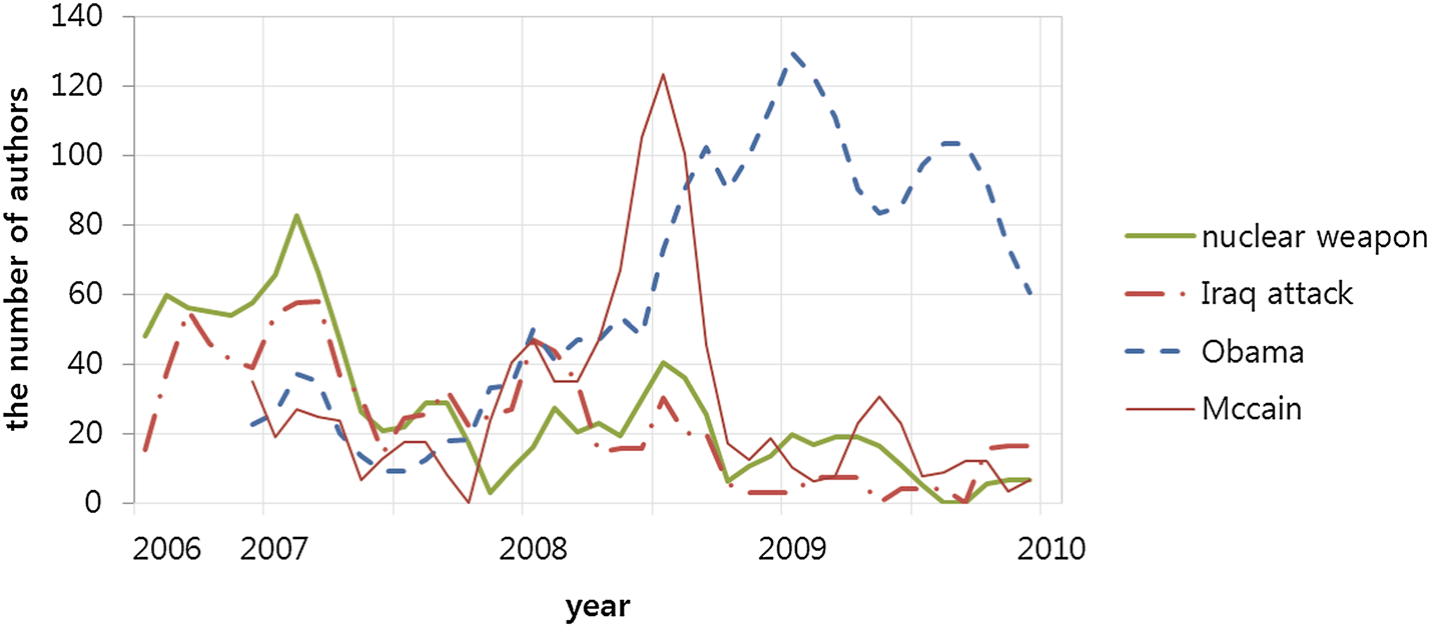
The time-series patterns of selected political topics

We observed major outbreaks in the time-series patterns of forum discussion on five topics: nuclear weapons, Iraq war, healthcare bill, McCain, and Obama. The keywords in selected topics are listed in Table 5 and their time-series are displayed in Fig. 7.

**Table 5:** The major topics and keywords in the US Politics Online forum

Topic group	Topic	Keywords
International issue	Nuclear weapon	Iran, nuclear, weapons, United States, Ahmadinejad, Russia
	Iraq war	Iraq, war, troops, Iraqi, military, forces, security, government
Domestic issue	Healthcare bill	Tax, healthcare, plan, pay, cost, insurance, income, program
Election issue	McCain	McCain, campaign, Palin, John, Governor, Presidential, Sarah
	Obama	Obama, president, Barack, presidential

**Fig. 8:**
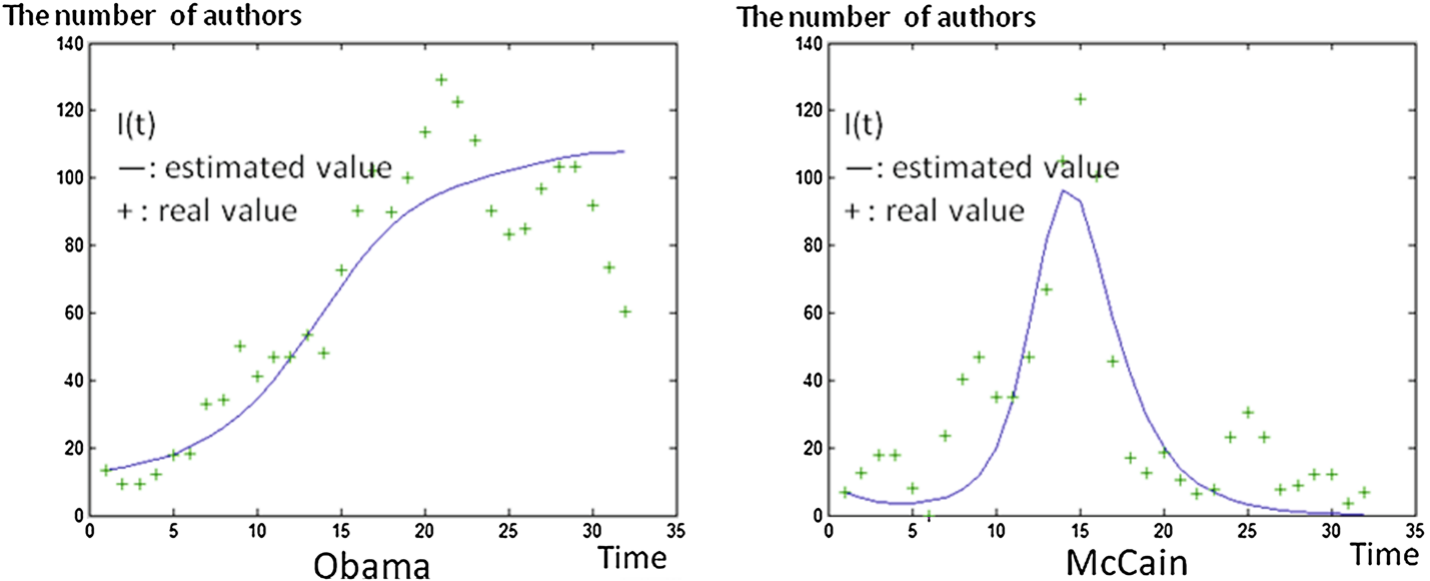
The estimated and real values of the SIR model on the topics of Obama and McCain

Figure 8 shows the estimated values of I(t) and the real values of the number of involved authors on the two president candidates. The estimated curves for the Obama-related topic in the left panel indicate that many authors are still discussing the topic, with infectives I(t) continuing to rise and the diffusion process is still happening; while the diffusion process for the McCain-related topic has ended as shown in I(t) of the right panel. John McCain was discussed more in the early stages, but discussion about him disappeared faster than discussion on Barack Obama (Table 6).

**Table 6:** Parameter estimation results on the political forum

Topic	MSE	$R^{2}$	S(0)	$\alpha$	$\beta$	$\mu$	K
Nuclear weapon	9.90E+03	0.4379	142.9	0.0076	0.9500	0.2670	931.8
Iraq war	9.27E+03	0.4739	166.4	0.0062	0.9115	0.2565	861.4
Healthcare bill	6.81E+02	0.5761	21.7	0.0180	0.5696	0.2995	208.7
Barack Obama	8.65E+03	0.7929	67.2	0.0039	0.2212	0.0937	1022.7
John McCain	8.20E+03	0.7190	140.4	0.0034	0.8078	0.2232	709.3

## Discussions

In this work, we tried to find out the underlying mechanism in the occurrence of the spiky discussion on a specific topic. Previous research that addressed the idea and opinion contagiousness formed a basis of this research. In a web forum, we could derive following observations. Users in the web forum react to others posts. It implies that users interact with each other through posts. Thus, we adopt the disease diffusion model that explains the disease outbreak through the contact between people. The aim of the diffusion model is to understand the mechanisms of the spread of new diseases, ideas and products, to predict success or failure of diffusion in the early stages, and to design strategy to increase or reduce the chances of diffusion. The time-series model also has an aim to forecasting future trend with current data; the time-series model is mainly based on the identification of common patterns of consecutive data points. While the time-series model is the absence of reasoning on diffusion process except examining data occurrence patterns, the diffusion model is based on the fact that diffusion happens mainly due to user interaction. In this work, we tested the feasibility of a baseline epidemic model to describing the topic diffusion in the web forum. Through the empirical tests, we presented the coefficient value, R-squared that indicates how well data fit a model and this is widely adopted in time-series modeling. Since the mathematical model, especially the deterministic model, simplifies the diffusion process, it does not provide a complete analysis on that. The purpose of the mathematical model is the description of the diffusion process and not a complete analysis. We also simplified the topic diffusion process without consideration forum characteristics except the contagion between people. This simplification enables us to build a theoretical model. The mathematical model does not provide individual-level knowledge such as who will be infected by a topic or when a user will be infected. However, the usefulness of a mathematical model is to obtain system-level measurements and test hypotheses using them. According to the estimation results, we found that the SIR model is a plausible model for the topic diffusion in the web forum. For major topics, we can say that 43 % of variances in time-series patterns are explained by the diffusion model at least because the lowest R-square value is 0.43. All topics that we tested exceed the lower bound of the moderate range. Thus, we claim that the topic diffuses among authors mainly by the interaction between them and thereby, it causes a peak of author participations. Heeler and Hustad (1980) addressed that the soundness of structural test guarantees the forecasting validity of the model. Colbaugh and Glass (2009) proved that very early dispersion of a diffusion process across network communities is a reliable early indicator that the diffusion will ultimately involve a substantial number of individuals with case studies involving emergence of the Swedish Social Democratic Party at the turn of the twentieth century, the spread of SARS in 2002–2003, and blogging dynamics.

In this work, we just showed the structural soundness of the baseline epidemic model over the topic diffusion in the web forum. However, after testing structural soundness, we can perform forecasting. Additionally, we can estimate how many authors have latent interest on each topic at the initial phase of the diffusion process. We can also estimate the expected duration and the intensity of diffusion process at an initial stage. Even though these estimation measurements become reliable when it closes to the peak, we can estimate them in an adaptive way. We can compare those values for topics that lead the peak. We also can predict the outbreak of topics through the examination on initial stage of diffusion process using the following broad principal of the mathematical epidemic model. If the average number of secondary infections caused by an average infective is <1 a disease will die out, while if it exceeds one there will be an epidemic (Diekmann et al. 1990). In the case when the diffusion process follows an epidemic model, this threshold behavior enables us to estimate the likelihood of an outbreak, which is a peak of the diffusion process. The most important usefulness of building a mathematical model is that we can incorporate the forum characteristics into the diffusion model. We can perform further investigation on other factors that may affect the diffusion dynamics by incorporating those factors into the model. For instance, the sentiment of posts may determine the infectivity of a topic. Then, we can design the model with the infective rate varying depending on the cumulated sentiment score of posts. Another possible hypothesis is that there exists the prey-decay mechanism in the web forum. It is a common sense that when new-coming influencing news come out, old news die out. We expect that same phenomena may occur in the web forum. When an influencing topic emerges, people’s interest may stop participate in a previous topic. The competing relationship between a new emerging topic and a previous topic can be incorporated into the proposed baseline model to test the above hypotheses. For a marketing purpose of this research, the marketer can use this model by identifying key words related to the marketing campaign. Using this model, the marketer can predict the outbreak and die-out of a marketing campaign and how long it lasts when it diffuses by examining the initial patterns of diffusion process. In a case when a new marketing campaign comes out, the market can find a similar one from previous campaigns and apply the parameters of the similar one for forecasting success or failure of a new marketing campaign. For a political perspective, the politician can examine which political-related topics are diffused contagiously. For example, during an election, based on the current diffusion patterns of key words related to candidates, we can infer who will win the election. Our work has following limitations. This system level model does not provide specific information about who will be infected. It just gives aggregated information how many users will react to a topic. Second, even though, we can use this model for forecasting for an emerging topic based on diffusion process of similar topics in past, but the reliability of forecasting becomes reliable when the point of forecasting reaches to the peak. Furthermore, identifying similar topics based on semantic similarity is not trivial and a new topic can generate totally different diffusion process from past topics even if it is similar to them semantically.

## Conclusions

We proposed an integrated and novel methodology to model opinion/idea diffusion in web forums. The SIR model, frequently used in previous research to analyze both disease outbreaks and knowledge diffusion, is adopted for the web forum. The model was evaluated on a large longitudinal dataset from the web forum of a major retail company and a dataset from a general political discussion forum. The experiment results revealed that the SIR model performed well in modeling topic diffusion in web forums. This research has two contributions. We extended the information diffusion research to a new domain: web forums. We also examined the possibility of applying the epidemic model to topic diffusion in web forums. For future research, we plan to apply an epidemic model to sentiment diffusion in web forums. The epidemic model with the two classes of positive and negative opinion would be fit to the sentiment diffusion. Consideration of the competency and interaction between positive opinion and negative opinion on a topic would also improve the modeling accuracy.
